# Editorial: Sensory control of posture and gait: integration and mechanisms to maintain balance during different sensory conditions

**DOI:** 10.3389/fnhum.2024.1378599

**Published:** 2024-03-05

**Authors:** Jana Kimijanová, Zdenek Svoboda, Jia Han

**Affiliations:** ^1^Center of Experimental Medicine, Slovak Academy of Sciences (SAS), Bratislava, Slovakia; ^2^Department of Natural Sciences in Kinanthropology, Faculty of Physical Culture, Palacký University, Olomouc, Czechia; ^3^College of Rehabilitation Sciences, Shanghai University of Medicine and Health Sciences, Shanghai, China; ^4^Research Institute for Sport and Exercise, University of Canberra, Canberra, ACT, Australia; ^5^Department of Nursing and Allied Health, Swinburne University of Technology, Hawthorn, VIC, Australia

**Keywords:** postural control, gait, sensory integration, sensory reweighting, elderly, neurological impairment

Dynamic balance is an essential component of human movement and requires a complex interplay of sensory inputs, neural processes, and musculoskeletal coordination. Specifically, information from proprioception, vision, and the vestibular system is organized to provide suitable motor actions via sensory integration and sensory-to-motor transformations that activate appropriate muscles. Responding effectively to a changing environment requires the dynamic adjustment of the contribution of different sensory inputs. This adaptation process, sensory reweighting, is crucial for maintaining postural stability during movement. Aging, as well as various neurological disorders, can significantly disrupt these intricate mechanisms, leading to functional impairments and thereby compromising the accuracy and precision of motor control. These disturbances often result in increased postural imbalance, affected gait patterns and a higher risk of falls.

The current Research Topic presents nine studies that focus on postural responses in different sensory conditions and analyse the mechanisms of sensory integration during standing and walking in healthy young adults, as well as in the elderly and patients with neurological diseases, aiming to improve balance or find ways of regaining postural stability.

The novelty of the studies arises from several aspects. Some of them observe healthy adults under more demanding conditions, such as simulated exposure to height in a virtual reality environment or treadmill-induced unpredictable slip disturbances. Other studies, focusing on different groups of patients, describe factors that can affect the control of posture and gait, including the integration of different sensory inputs. Further studies provide new knowledge related to the verification of new tools and procedures. Finally, some studies explore the relationships between postural control and specific intervention or training.

Two articles in this Research Topic provide insight into the assessment of dynamic balance in conditions with increased postural threat, modifying proprioception and risking the loss of balance, thus requiring adequate postural recovery. Bzdúšková et al. investigated the effect of height exposure in a virtual environment with an intense threat of vibration-induced falling while standing on an uneven surface. A more stiffened posture was observed with a fear of height, and they suggested that fear significantly modulates protective postural adjustments under threat on multiple levels, including fear-related visual dependence, increased gain of vestibular inputs, increased muscle spindle sensitivity, and more conscious balance control. Lee et al. explored reactive recovery responses to unpredictable slip perturbation during gait and the effect of vibrotactile cueing. They confirmed that vibrotactile cueing significantly improved gait kinematics during the recovery period following an unpredictable slip, while the activity of some subregions of the prefrontal cortex substantially increased during this period. These results can significantly contribute to the development of novel strategies that prevent falls or alert individuals to the risk of falling.

The most falls-prone individuals are the elderly and those suffering from neurological disorders, where damage to nerve fibers can cause sensitivity impairments, leading to postural instability. Three articles in this Research Topic focused on sensorineural integration and its deficits in patients with impaired balance due to neurological impairment. Walz et al. captured whole-body movements during the Timed Up and Go test. They revealed down-scaled mean joint velocities in patients with polyneuropathy compared to healthy individuals and assumed that this overall slowing was caused by the reduced quality of proprioceptive signals. Therefore, they suggested that these patients scaled their postural control strategy commensurate with the quality of the sensory signals. Drawing from their previous studies, they argue that postural behavior and sensory reweighting in these patients are modifiable by exercise intervention. The effect of exercise intervention via 10 days of intensive treadmill training in patients with neurological impairment was investigated by Dalin et al., who examined changes in postural abnormalities after training in patients with hereditary spastic paraplegia, which is also characterized by peripheral neuropathy. They confirmed the benefits of treadmill training by means of learning to compensate for functional deficits. Their model-based analysis of sensorimotor behavior was capable of differentiating between functional correlates of a disease's anatomical substrate and the parameter changes due to therapeutic interventions. Next, Awosika et al. characterized sensory reweighting in a cohort of chronic stroke survivors suffering from residual postural instability and walking impairment. Their study is the first to report post-stroke sensory reweighting insufficiency and its consequential influence on walking capacity, highlighting an increased reliance on vision and proprioception. These studies emphasize incorporating multisensory system integration testing and strengthening into the rehabilitation of neurological patients.

The subsequent contributions in this Research Topic propose novel techniques for the assessment of sensory reweighting mechanisms. Zur et al. validated the short version of their balance test that uses head movements for improved and more sensitive postural testing by employing precise kinetic measurements to reliably detect even minimal differences in postural control. They also reported normal physiological ranges of the Zur Balance Score in healthy participants. The importance of assessing the impact of head movements on postural stability, especially in people who might have impaired vestibular function, was emphasized by Shiozaki et al., who examined the effect of a dynamic balance task on changes in lateral vestibulospinal tract (LVST) excitability. As LVST significantly contributes to balance, postural assessment during a dynamic task along with galvanic stimulation may provide a better estimate of postural control, with an emphasis on the vestibular system. They proposed that increased LVST excitability may be associated with improved balance. Given the crucial role of vision in postural control, the ability to detect self-motion from optical flow is of great importance. Motivated by the lack of visual motion detection thresholds measured in conditions with natural head movement, DiBianca et al. investigated new possibilities to determine these thresholds using a virtual visual environment. They confirmed that visual motion detection thresholds can be reliably measured during standing and walking with higher threshold values during walking. This finding opens new horizons for exploring the relationship between visual motion detection thresholds and falls risk in the elderly and other vulnerable populations. Finally, Viseu et al. explored the specificity of sports practice on the sensory control of balance and noted that only specific directionally oriented variables are able to reveal the effects of sports practice on postural control. Selecting relevant variables may be beneficial for the follow-up assessment of athletes or patients to observe the effects of training or therapeutic intervention.

In conclusion, this Research Topic provides valuable insights into the mechanisms of sensory integration within balance maintenance in both healthy individuals and neurological patients. The articles highlight the importance of accurate measurements of sensory reweighting processes and suggest that interventions targeting all sensory systems may be effective in improving impaired postural control. Overall, the research has significant implications for the development of new interventions and approaches to enhance postural control, potentially reducing the risk of falls.

## Author contributions

JK: Writing – original draft. ZS: Writing – review & editing. JH: Writing – review & editing.

